# Extended fMRI-Guided Anodal and Cathodal Transcranial Direct Current Stimulation Targeting Perilesional Areas in Post-Stroke Aphasia: A Pilot Randomized Clinical Trial

**DOI:** 10.3390/brainsci11030306

**Published:** 2021-02-28

**Authors:** Leora R. Cherney, Edna M. Babbitt, Xue Wang, Laura L. Pitts

**Affiliations:** 1 Think + Speak Lab, Shirley Ryan AbilityLab, Chicago, IL 60611, USA; ebabbitt@sralab.org (E.M.B.); laura.pitts@uni.edu (L.L.P.); 2 Department of Physical Medicine and Rehabilitation, Feinberg School of Medicine, Northwestern University, Chicago, IL 60611, USA; 3 Department of Communication Sciences and Disorders, Northwestern University, Evanston, IL 60208, USA; 4 Department of Radiology, Feinberg School of Medicine, Northwestern University, Chicago, IL 60611, USA; xuewang2@gmail.com; 5 Department of Communication Sciences and Disorders, University of Northern Iowa, Cedar Falls, IA 50614, USA

**Keywords:** transcranial direct current stimulation (tDCS), aphasia, speech language therapy, noninvasive brain stimulation (NIBS), nonfluent, interhemispheric inhibition (IHI)

## Abstract

Transcranial direct current stimulation (tDCS) may enhance speech and language treatment (SLT) for stroke survivors with aphasia; however, to date, there is no standard protocol for the application of tDCS in post-stroke aphasia. We explored the safety and efficacy of fMRI-guided tDCS on functional language and cortical activity when delivered to the lesioned left hemisphere concurrently with SLT across an extended, six-week treatment period. Twelve persons with chronic, nonfluent aphasia following a single left-hemisphere stroke participated in the three-arm (anodal vs. cathodal vs. sham) single-blind, parallel, pilot trial. No serious adverse events occurred during 30 treatment sessions or in the following six weeks. All groups demonstrated functional language gains following intensive treatment; however, active tDCS resulted in greater gains in standardized, probe, and caregiver-reported measures of functional language than sham. Evidence declaring one polarity as superior for inducing language recovery was mixed. However, cathodal stimulation to the lesioned left hemisphere, expected to have a down-regulating effect, resulted in increased areas of cortical activation across both hemispheres, and specifically perilesionally. Generalization of these preliminary findings is limited; however, results are nevertheless compelling that tDCS combined with SLT can be safely applied across extended durations, with the potential to enhance functional language and cortical activation for persons with aphasia.

## 1. Introduction

Approximately one-third of the 104 million stroke survivors worldwide live with resultant aphasia, or difficulty comprehending and/or expressing language [1,2]. Persons with aphasia often experience significant secondary disability including depression, isolation, decreased quality of life, and difficulty resuming everyday life activities including returning to work [3,4,5,6,7]. Despite advances in gold-standard behavioral speech-language therapy (SLT), the recovery of functional communication is often slow with minimal to moderate gains [8,9]. Transcranial direct current stimulation (tDCS) may bolster aphasia recovery when combined with SLT; however, optimal parameters for tDCS protocols to rehabilitate aphasia remain unclear due to differences across studies in research designs, tDCS protocols, and participant characteristics [10,11,12,13]. The current evidence is insufficient to (1) determine which polarity (anodal, cathodal, or dual) and stimulation site is most efficacious, (2) establish safety across extended use, and (3) fully characterize the impact of adjuvant tDCS on functional language outcomes [10,11].

In tDCS, a mobile battery-operated direct current stimulator delivers a constant, weak direct current between two surface electrodes on the scalp. One electrode is placed on the site overlying the cortical target and a reference electrode is placed over an area considered to be remote from the cortical target such as the contralateral supraorbital area, contralateral mastoid, or contralateral shoulder. The nature of the effect depends on the polarity of the current, i.e., the direction of the flow of the current. In general, it is assumed that anodal tDCS results in increased cortical excitability whereas cathodal tDCS decreases cortical excitability [14,15,16].

Across the aphasia literature, anodal stimulation (A-tDCS) to the lesioned hemisphere has been most often investigated with the intent of promoting perilesional activation whereas cathodal stimulation (C-tDCS) has been applied to the intact, homologous cortex to downregulate activity largely based on the theory of interhemispheric competition [10,17,18]. However, there is growing concern that suppressing interhemispheric inhibition (IHI) in the contralesional hemisphere may be an oversimplified or erroneous model of functional reorganization after a stroke [18].

Studies investigating cathodal stimulation to the lesioned left hemisphere are rare. Interestingly, the first investigation of tDCS in aphasia rehabilitation reported improved naming accuracy following a single session of cathodal stimulation over the left frontotemporal cortex (without concurrent SLT) across eight stroke survivors with non-fluent aphasia [19]. More recently, cathodal (C-tDCS) stimulation to the lesioned hemisphere proved to be the most consistent montage to immediately increase naming accuracy when compared to a sham condition and additional permutations of either A-tDCS or C-tDCS to each hemisphere [20]. However, to our knowledge, there have been no other reports of cathodal stimulation to the left lesioned hemisphere in persons with aphasia.

In addition to decisions regarding selection of the cortical hemisphere and the polarity of stimulation, there is insufficient information about the specific stimulation target. In many studies, the stimulation site is selected apriori and identified using the 10–20 EEG measurement system in the absence of neuroimaging data. However, selecting a common site for all participants without knowledge of a participant’s specific lesion location and size may result in electrodes being placed on tissue that is not viable, thereby altering the flow of the current and subsequent effects of the tDCS [21,22]. Other studies have completed imaging prior to tDCS to ensure that the electrodes are placed over surviving viable tissue [23], but have not individualized the electrode location based on the functional integrity of the tissue. Selecting a stimulation site based on the individual’s specific anatomic and functional neuroimaging data may lead to more consistent results within and across studies [12]. Thus, the present heterogeneity of applied montages across investigations precludes the development of guidelines for tDCS applications to treat post-stroke aphasia.

Furthermore, a recent 2019 Cochrane Review of tDCS applications in post-stroke aphasia rehabilitation concluded that although no serious, harmful effects of tDCS have been published, further trials are needed to determine the safety of tDCS, especially when applied over extended durations such as those similar to clinical intervals of SLT [10]. The same Cochrane Review and others also noted that there is no current consensus that tDCS may enhance aphasia recovery beyond improved accuracy in naming nouns [10,13]. Indeed, the majority of studies have paired the tDCS with some form of naming therapy that is not always evidence-based [13,24]. Thus, a critical question remains: Does adjuvant tDCS have any therapeutic use in aphasia rehabilitation aside from pairing with single word naming treatments, especially without consensus for any clinically meaningful gains in functional language?

Here, we explored the effects of an extended, six-week application of perilesional A-tDCS, C-tDCS, or sham (S-tDCS) stimulation with concurrent SLT on language skills and cortical activation maps in nonfluent aphasia. The specific aims were to assess whether prolonged administration of tDCS is safe, and if a single polarity targeting the lesioned left hemisphere improves and/or maintains gains in language to a greater extent than sham stimulation and the opposing polarity. Results may substantially shift paradigms that most often perpetuate anodal and largely ignore cathodal stimulation to the lesioned hemisphere. Data supporting extended tolerability and potential for functional language gains will reinforce the promise of feasibility and efficacy of adjuvant tDCS applications in routine aphasia rehabilitation.

## 2. Materials and Methods

This Phase 1 single-blind placebo-controlled randomized clinical pilot study was approved on 5/8/2009 by the Institutional Review Board at Northwestern University (STU00011132) and subsequently allocated participants across three parallel arms with an allocation ratio of 1:1:1 (A-tDCS: C-tDCS: S-tDCS). The study was conducted in accordance with the Declaration of Helsinki and registered as a clinical trial (NCT01486654). All subjects provided informed consent prior to participation. No interim analyses or stopping guidelines were enacted or required by protocol.

### 2.1. Participants

Participants were enrolled from May 2010 to December 2012 at an urban rehabilitation center. Inclusion criteria were as follows: men or women with a diagnosis of non-fluent aphasia subsequent to a single-event, unilateral left hemisphere stroke that occurred more than six months prior to participation and was confirmed by CT scan or MRI; an Aphasia Quotient on the Western Aphasia Battery-Revised (WAB-R AQ) of 25–75 [25]; premorbidly literate in English; right-hand dominant as determined by the Edinburgh Handedness Inventory [26]; at least an eighth grade education; current visual acuity no worse than 20/100 corrected in the better eye; auditory acuity no worse than 30 dB HL on a pure tone hearing screening (conducted at 500, 1000, 2000, and 4000 Hz), aided in the better ear; seizure-free for six months or longer prior to study enrollment. Participants were excluded if they had a neurological condition other than cerebral vascular disease that could potentially affect cognition or speech (e.g., Parkinson’s disease, Alzheimer’s dementia, traumatic brain injury); significant psychiatric history (e.g., severe depression or psychotic disorder requiring hospitalization); or contraindications for magnetic resonance imaging (MRI). A sample size of 12 participants (four in each arm) was considered to be feasible given the exploratory nature of the study and its financial and time constraints.

### 2.2. fMRI-Guided Localization of Perilesional Stimulation Site

Structural and functional scans were obtained on a Siemens 3T TIM Trio scanner (Siemens Medical Solutions, Erlangen, Germany) with a 32-channel head coil at two time points: before and after six weeks of SLT paired with tDCS. Pre-treatment scans were used to determine eligibility, establish baseline patterns of brain activation during language tasks, and guide the identification of individualized stimulation sites for tDCS. Post-treatment scans assessed physiological changes associated with treatment. T1-weighted anatomical images were acquired with TR/TE of 2300/2.97 ms, flip angle of 9°, matrix resolution of 176 × 256 × 256, and voxel size of 1 × 1 × 1 mm. Functional tasks were acquired with high-resolution gradient echo-planar imaging (EPI) data, TR/TE of 2200/20 ms, flip angle of 80°, in-plane resolution of 128 × 116, voxel size of 1.72 × 1.72 × 3 mm across a total of 37 slices for whole brain coverage. Tasks included: 1. semantic categorization, 2. oral reading of a word within a sentence, and 3. imitation of consonant-vowel syllables. All three tasks were pre-trained within three days prior to the fMRI evaluation, then retrained just prior to entering the MRI.

Task 1 involved a semantic decision. Participants were shown two words aligned vertically on the screen. The top word was a category name (e.g., flower) and directly underneath was an object label (e.g., either rose or chair). Using their unimpaired, left hand, participants pressed one of two buttons on a fiberoptic device shaped like a computer mouse to indicate either “yes” or “no” regarding whether or not the object label belonged to the above category. Each pairing was displayed for up to five seconds or until a response was made. Task 1 included eight blocks (i.e., 48 s in duration with eight pairs of words presented in random order) interleaved with eight rest blocks (i.e., 48 s in duration during which the participant was asked to focus on a cross displayed in the middle of the screen). The total time for Task 1 was 10 min, 25 s.

Task 2 required participants to read aloud single words (i.e., oral reading). Participants were shown three- to five-word sentences on the screen for a total of six seconds each. For half of the presented sentences, one of the content words changed from black font to red font after two seconds and stayed red for the remaining four seconds. For the other half of the presented sentences, all words remained in black font while displayed for six seconds. Participants were asked to read a single word aloud only if it turned red and to remain silent if no color changes occurred. During Task 2, there were six overt speech blocks interleaved with six covert blocks. Each block contained four short sentences. The total time for Task 2 was 8 min, 40 s.

During Task 3, participants watched short, 1.5 second video clips of a woman producing consonant-vowel syllables (e.g., pa, fa, ta, and θa) and were required to imitate the syllables. Task 3 was an event-related design with jittered intertrial intervals. The total time for Task 3 was 10 min, 39 s.

These fMRI tasks were selected because they required skills necessary for the behavioral treatment provided concurrently with the tDCS (i.e., matching words, reading aloud words within sentences, and observation and imitation of oral motor movements). It was expected that these fMRI tasks would subsequently recruit cortical regions required for the behavioral treatment such as premotor cortex (including the pars opercularis of the inferior frontal gyrus), superior temporal gyrus and occipito-temporal cortex—regions thought to be particularly important in speech production, language comprehension and reading, respectively [27,28]. Furthermore, these tasks had been used in previous studies with aphasia, including one study where common areas of cortical activation in the left hemisphere were used successfully to identify the site for epidural cortical stimulation [27,28,29].

**Figure 1:**
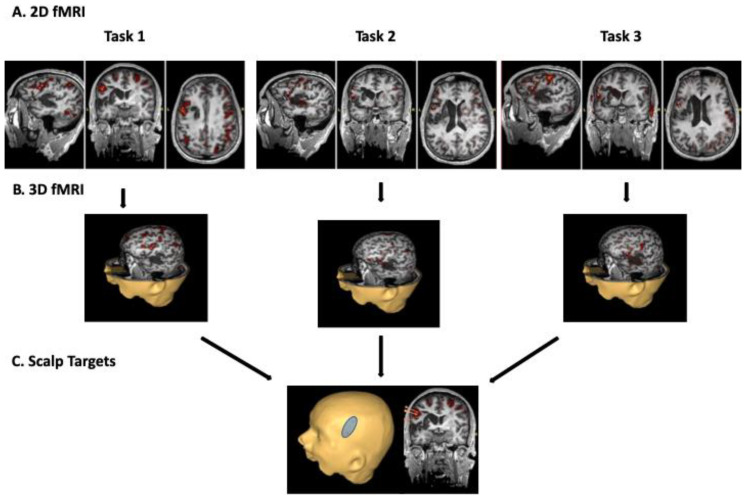
Individualized stimulation site was determined based on overlapping BOLD signals in the left hemisphere across three speech–language tasks. In each image, orange markers were placed using the neuronavigation system to identify locations of cortical activation. fMRI BOLD signals for each task were overlaid onto 2D images (**A**) and a 3D head reconstruction (**B**). While it is impossible to encompass all areas of activation, care was taken to locate stimulation sites with demonstrated activation in at least two of the three tasks. Lastly, scalp projections (**C**) of each stimulation site were generated and verified to fit underneath the surface area of the electrode.

**Figure 2:**
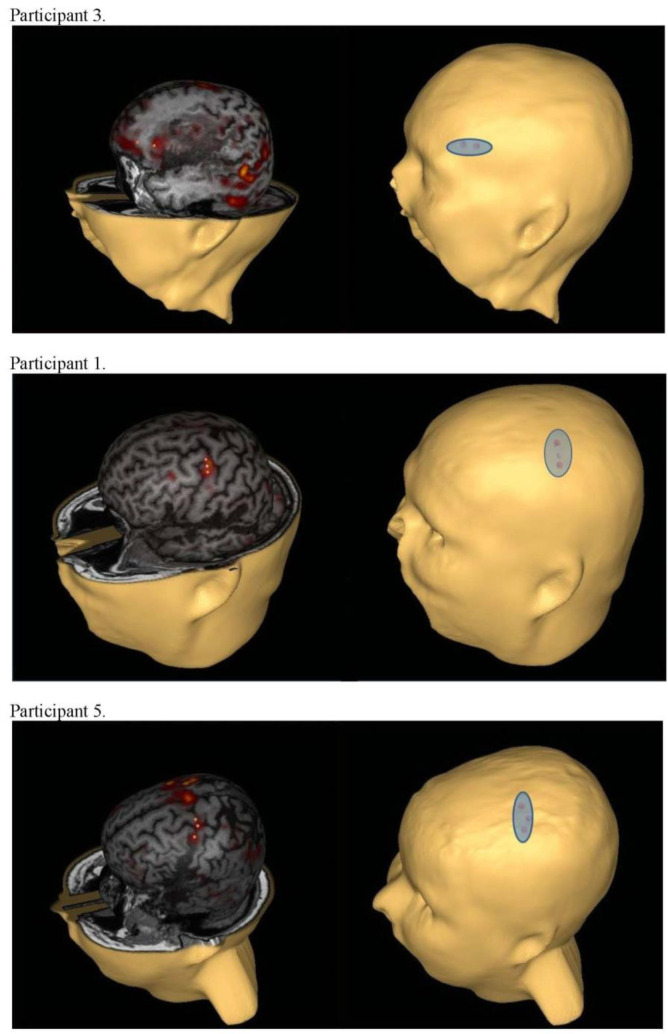
Examples of fMRI-navigated, perilesional stimulation sites across three participants. The figure illustrates how the fMRI “hot spots” were incorporated into one electrode location with the proper orientation. For Participant 3, the electrode placement was based on activation in tasks 2 and 3. For Participant 1, the electrode site incorporated activity on all three fMRI tasks. For Participant 5, electrode placement was specific to tasks 1 and 2. Thus, electrode placement was individualized for each participant. The reference electrode was placed horizontally over the right contralateral supraorbital area for all participants.

**Figure 3:**
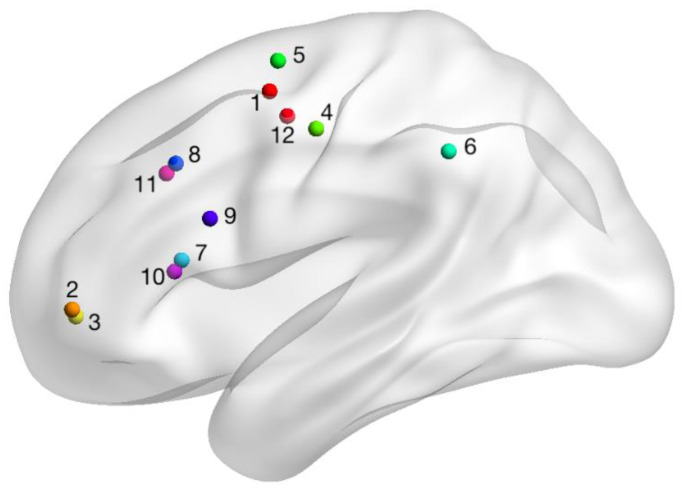
Plotted fMRI-navigated stimulation sites for all study participants on a model of a standard left hemisphere. Numbers correspond to the participant number (Table 1).

The stimulation site, or fMRI-navigated electrode placement, was defined a priori and determined as overlapping left hemisphere areas of cortical activation (i.e., BOLD signal) across at least two of the three tasks for each individual. fMRI images were overlaid onto 2D MR cross-sectional images and a 3D reconstructed head image, and co-registered with external cranial landmarks to generate a scalp projection using the eXimia neuronavigation system (Nexstim Ltd., Helsinki, Finland). The scalp projection of the stimulation site was marked on the participant with indelible ink, which was maintained throughout the treatment interval and calibrated between the neuronavigation system and manual tape measurements using external landmarks (e.g., tragus of the outer ear, nasion). For all participants, periodic validation of electrode placement was conducted within the first two weeks of treatment using the neuronavigation system. Figure 1 illustrates the process of localizing the fMRI-guided stimulation site using the neuronavigation system. Figure 2 illustrates examples of perilesional electrode location and orientation for three participants (i.e., the first participant randomized to each arm). The orientation of the active electrode was selected so that the greatest area of activation was covered. Figure 3 illustrates the fMRI-guided stimulation sites for all study participants plotted in standard Montreal Neurological Institute (MNI) space on a normal model of the left hemisphere. Table 1 reports each individual’s stimulation site by Harvard–Oxford Brain Atlas classifications. For all participants, the reference electrode was placed horizontally over the right contralateral supraorbital area.

### 2.3. Randomization and Blinding

Participants were randomized to one of three treatment arms (A-tDCS, C-tDCS, or S-tDCS); each arm was delivered simultaneously with computerized SLT. Participants were randomized after all baseline assessments were completed and the stimulation site had been determined. Randomization was generated in advance by a statistician who provided the principal investigator (LRC) with individual, sequentially numbered sealed envelopes containing the randomization for prospective participants. Randomization was blocked for every three eligible participants and stratified by aphasia severity (i.e., a cut-off score of 55 on the WAB-R AQ). The principal investigator, who was not involved in recruitment, assessment, or treatment, then allocated the envelopes. Participants, care providers/family members, and the evaluator remained blinded to group assignment throughout the study. In the sham treatment arm, stimulation was provided in a programmed, ramp-like fashion for 30 s, and then shut off. The parameters for the sham stimulation were chosen based on published reports that perceived that sensations from tDCS (e.g., tingling on the skin) are transient and fade after the first 30 s of stimulation [30]. Therefore, participants who received sham tDCS perceived physical sensations of tingling that were similar to the sensations perceived by participants who received tDCS for 13 min. To confirm proper blinding of sham versus active tDCS, following completion of the post-treatment assessment, participants were asked to guess whether they had received active or sham stimulation, even though treatment allocation remained undisclosed. The four participants who received sham tDCS all indicated that they thought they had received active stimulation. Of the eight participants who received active stimulation, only one participant (#2) indicated that he thought he had received sham stimulation.

### 2.4. tDCS Intervention

tDCS was delivered using a constant current stimulator (Dupel Iontophoreis System, Empi, MN) via an 8 cm^2^ oblong (2 × 4) saline-soaked sponge electrode placed directly over the established stimulation site (Dupel BLUE, MN). A self-adhesive carbonized reference anode (48 cm^2^) was placed on the forehead, directly above the contralesional orbit.

Treatment sessions lasted 90 min and were completed five days a week for six weeks, for a total of 30 sessions. Across all study arms, 1mA of active (anodal or cathodal) or sham tDCS was applied to the lesioned left hemisphere during the first 13 min of the 90-minute speech–language treatment session. The parameters for active tDCS were strategically chosen to induce and prolong current-driven increases in cortical excitability, lasting up to 90 min post-stimulation [14,15,16]. Nitshe and colleagues have demonstrated that after short, 5- or 7-minute applications of low-intensity A-tDCS or C-tDCS (1mA) to the upper limb motor cortex, the amplitude of motor-evoked potentials (MEPs) quickly returned to baseline within a few min after stimulation had ended [14,15,16]. However, 13 min of A-tDCS at the same low-level intensity produced extended periods of elevated MEPs (i.e., approximately 150% of baseline), which were well-maintained up to 90 min post-stimulation [15]. Careful consideration of tolerability and safety was also reflected in the decision to apply a current intensity of 1mA across the longer duration of 13 min in the present study [31,32].

The stimulation was initially increased in a programmed, ramp-like fashion over several seconds until reaching 1 mA for all participants. Within both A-tDCS and C-tDCS arms, the stimulation was then maintained for a total of 13 min; however, in the sham condition, the stimulation was turned off after 30 s, outside of the view of the participant to maintain blinding. Additionally, a timer was set for 15 min after the start of the SLT, at which time the electrode and tDCS leads were removed for all participants prior to completing the remaining 75 min of computerized SLT.

### 2.5. Concurrent Language Intervention

**Figure 4:**
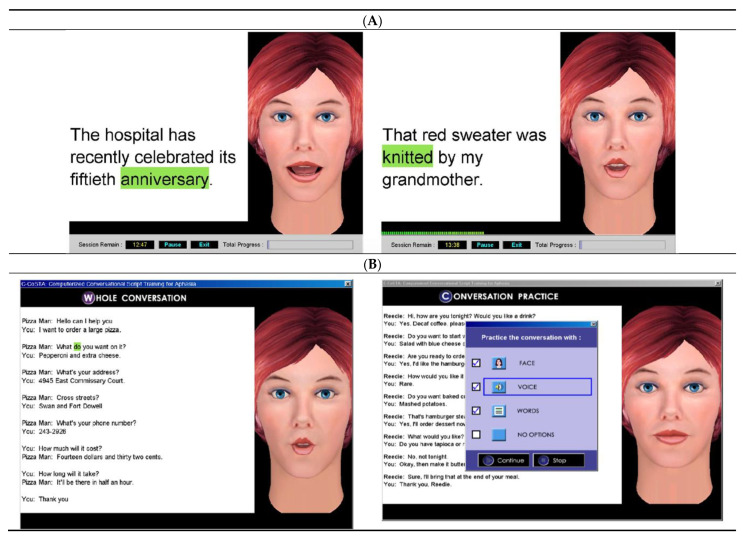
Sample screenshots showing the “virtual therapist” and examples of sentence and script stimuli in (**A**) AphasiaScripts^®^ and (**B**) ORLA^®^. Words are highlighted in green as the virtual therapist reads the sentences aloud by herself and then in unison with the participant. When the participant reads the sentences aloud in unison and independently, the green highlighting of the words and the virtual therapist’s mouth movements provide cues that help with the accuracy and rate of production of the words and sentences. In AphasiaScripts^®^, these cues can be removed one at a time during the conversation practice.

**Figure 5:**
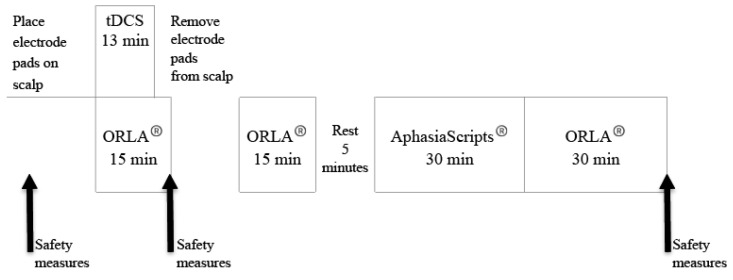
Schematic of a daily treatment session showing the sequence and length of time of each of the behavioral treatments: Oral Reading for Language in Aphasia (ORLA^®^) and AphasiaScripts^®.^ The tDCS was always applied with ORLA^®^. The schematic also shows when safety measures were collected.

Concurrent SLT intervention consisted of two, evidence-based treatment protocols that provide practice in auditory and reading comprehension of sentences and short scripts as well as production of functional language. Both treatments were administered via computer with a “virtual therapist” to ensure treatment fidelity across sessions and participants. Having two treatments served to increase participant engagement by breaking up any monotony that might occur during the lengthy 90-minute treatment session. First, participants received two sequential 15-minute intervals of Oral Reading for Language in Aphasia (ORLA^®^). The first 15 min of ORLA^®^ was always paired with the tDCS. With ORLA^®^, the participant repeatedly practices reading aloud sentences, first in unison with the “virtual therapist” and then independently [33,34,35]. Participants with WAB-R AQ scores less than 55 practiced sentences that were 3–5 words long, whereas those with WAB-R AQ scores greater than 55 practiced sentences that were 8–10 words long. The sentence stimuli for each ORLA^®^ treatment session were randomly selected by the computer from a pool of 150 sentences that were available at each level (i.e., 3–5 word level and 8–10 word level). Following a five-minute rest break, the participant then completed a 30-minute interval of AphasiaScripts^®^, which also incorporates reading aloud of sentences, but now embedded within scripted conversations [36,37,38]. Two sets of six scripts each were available, one set of shorter scripts with grammatically simple short sentences and another set of longer scripts with more complex sentences. Similar to ORLA^®^, the participant’s WAB-R AQ was used to determine which set of scripts was selected since overall script complexity of each set was similar to the complexity of the two levels of ORLA^®^. However, sentence stimuli practiced during AphasiaScripts^®^ were different from those practiced during ORLA^®^. Each script was practiced for five days, with a new script introduced at the beginning of each week of treatment. The last 30-min interval of treatment was continued reading aloud practice via ORLA^®^. Sample screenshots of AphasiaScripts^®^ and ORLA^®^ are included in Figure 4. A schematic of the procedures within a single, daily treatment session are illustrated in Figure 5. Participants did not receive any other individual or group aphasia treatment during their participation in the study, including the six-week follow-up period.

### 2.6. Outcome Measures

Outcome measures of safety and tolerability included vital signs of temperature, heart rate, and blood pressure. Vitals were completed using digital sensors at three time points during each session: before and after the tDCS interval and the end of the 90-minute treatment session. In addition, self-reported side effects were obtained at the same three time points using an aphasia-friendly questionnaire with an attached 10-point scale (where 10 represents the highest degree of discomfort). The same questionnaire was used during weekly follow-up phone calls to the participants throughout a six-week maintenance phase to document and address any long-term side effects from the tDCS.

The primary language outcome measure was the AQ of the WAB-R which has been identified as a core outcome measurement instrument for aphasia to be used in Phase I-IV clinical trials [25,39]. The Language Quotient (LQ) of the WAB-R which encompasses additional reading comprehension and written expression skills and the Communication Effectiveness Index (CETI) [40] served as secondary language outcomes. The CETI measures functional communication skills as reported by a caregiver. Caregivers were blinded to treatment allocation as well as patient performance during assessment and scoring of language outcome measures. The aforementioned language measures were administered before and after the six weeks of treatment and at six weeks following the end of treatment (i.e., follow-up). A gain of 5 points or greater on the WAB-R AQ and LQ was considered to be clinically significant, whereas a gain of 10 points or greater was considered to be clinically significant on the CETI [40,41].

Behavioral probe measures of oral reading accuracy and rate (words per minute, wpm) were also obtained at each assessment (pre-treatment, post-treatment, and follow-up) as well as weekly during the treatment interval. Probe tasks, including baseline probes, required the participant to read aloud ten sentences, randomly selected by the computer program from the pool of 150 trained ORLA^®^ stimuli. The participant’s sentence productions were scored and rated for percent accuracy and words per minute (wpm) according to the Naming and Oral Reading for Language in Aphasia 6-point scale (NORLA-6) [42]. Fifteen percent of all probes were randomly selected and checked for point-to-point inter-rater reliability of scoring between the treating clinician and an independent evaluator not associated with the study. Inter-rater reliability was 96.9% for accuracy and 97.4% for rate.

Neurophysiological changes were quantified through the number of activated voxels within a 5mm area around the lesion (perilesional cortex) and the ratio of activated voxels between the entire left vs. right hemisphere (left/right ratio) for each fMRI task and compared between pre- and post-treatment.

No changes were made to the outcomes after the trial commenced.

### 2.7. Analyses

For the primary language outcome measure, the WAB-R AQ, inferential analyses were conducted in SPSS 24.0 with post-hoc power analyses conducted with G*Power 3.1.9.7. Significance level was set at *p* < 0.05. A one-way ANOVA explored significant differences among the groups in baseline WAB-R AQ performance. Paired *t*-tests explored significant post-treatment changes in WAB-R AQ scores within each group. A mixed-model, two-way ANOVA was conducted to explore changes in WAB-R AQ at post-treatment by treatment group. Effect sizes for ANOVA and *t*-test results were calculated using (partial) eta squared (η2) and Cohen’s *d*, respectively, and interpreted based on Cohen (1988). As an exploratory pilot study with small sample size, there is risk for not detecting a true difference in outcomes across treatments due to lack of power. Thus, a post-hoc power analysis based on the observed difference among WAB-R AQ group means was conducted to estimate the sample sizes necessary for future studies to detect statistical differences among these treatment arms.

Group means and individual change scores on the standardized language measures (WAB-R AQ, WAB-R LQ, and the CETI) were also descriptively analyzed in relation to established benchmarks of clinical significance [40,41,43].

NORLA-6 performance varied greatly across participants at baseline. Therefore, NORLA-6 performance from (1) pre- to post-treatment and (2) pre-treatment to follow-up was calculated as a percent of gain for both individual participants and by group. Percent gain from pre- to post-treatment was calculated as the mean of three post-treatment probe scores minus the mean of three pre-treatment probe scores divided by the mean of the three pre-treatment probe scores. The percent gain from pre-treatment to follow-up testing was calculated similarly as the mean of the two follow-up probe scores minus the mean of the three pre-treatment probe scores divided by the mean of the three pre-treatment probes scores. Effect sizes for NORLA-6 gains by individual participants were calculated using Busk and Serlin’s *d* statistic with reference to established benchmarks for a small (2.6), medium (3.9), or large (5.8) effect size that were derived from meta-analyses of aphasia treatments [44,45,46]. A priori determination of a clinically significant effect size was greater than 3.9 (medium effect).

In regard to exploring changes in cortical activation patterns, the duration of each fMRI task varied slightly with 284 volumes collected during Task 1, 230 volumes during Task 2, and 285 volumes during Task 3. The first six volumes of each run were discarded to allow the MRI signal to reach equilibrium. SPM8 (http://www.fil.ion.ucl.ac.uk/spm/software/spm8/) was used to analyze images. Functional images were realigned with the first volume, re-sliced, co-registered to the T1 anatomical image, spatially smoothed with a 6 mm full-width at half-maximum Gaussian kernel, and high-pass filtered (cutoff period of 256 s for Tasks 1 and 2 and 128 s for Task 3. Fixed effect analyses were conducted on each task using a generalized linear model. Each acquisition block was modeled independently and convolved with the canonical hemodynamic response function combined with time and dispersion derivatives. Individual activation maps (F-contrast) for Tasks 1 and 3 were contrasted between baseline and task. For Task 2, active maps were contrasted between overt and covert conditions. The number of and ratio of activated voxels was counted for both left and right hemispheres using the functional Magnetic Resonance Imaging of the Brain Software Library (FSL) image analysis suite’s FSLSTATS program (http://www.fmrib.ox.ac.uk/fsl/) [47]. The manually segmented lesion masks based on anatomical images were spatially transformed to the individuals’ functional space using the FLIRT toolbox (part of FSL) [48,49,50]. Perilesional masks were created by dilating the lesion masks using a 4 mm Gaussian kernel. Activated voxels within the perilesional masks on the left hemisphere were obtained to indicate the level of perilesional brain activation.

## 3. Results

**Figure 6:**
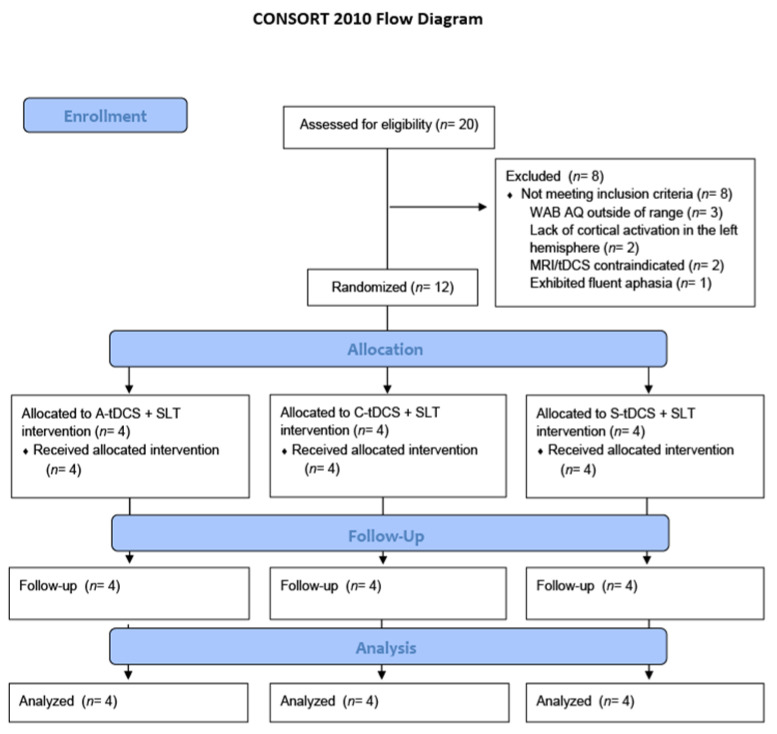
CONSORT 2010 Flow Diagram. Note. A-tDCS = anodal, C-tDCS = cathodal, and S-tDCS = sham stimulation. WAB-R AQ = Western Aphasia Battery-Revised Aphasia Quotient.

Twenty participants were screened with 12 participants enrolled in the study. A CONSORT flow diagram is included as Figure 6; Table 1 reports demographics and stimulation site for enrolled participants. Eight participants were excluded as follows: three participants had WAB-R AQ scores outside of target range, two participants did not demonstrate cortical activation in the left hemisphere across the fRMI tasks, two participants exhibited MRI/tDCS contraindications, and one participant exhibited fluent aphasia during pre-treatment assessment. We note that Participant 12 was enrolled even though he scored slightly below the WAB-R inclusion cut-off score of 25 because the study support was ending; this was approved by the IRB.

All participants completed the 30 treatment sessions including the monitoring of vital signs and adverse events. All participants completed weekly follow-up phone calls during the six-week follow-up interval after the conclusion of treatment. Participants also completed primary and secondary measures of language outcomes, except for Participant 8, who did not have a significant other available to complete the CETI and, therefore, CETI scores are missing. In addition, during the follow-up assessment, Participant 8 declined to continue with follow-up testing after the administration of the WAB-R AQ; thus, the remaining follow-up data points are missing.

### 3.1. Safety and Tolerability of Prolonged tDCS

Vitals were stable and remained within normal limits during the six weeks of treatment for all participants. No serious adverse events were reported across the treatment interval, in weekly phone calls between the end of treatment and the six-week follow-up assessment, or at follow-up appointments. Table 2 shows the frequency of side effects (tingling/itching under the electrodes, fatigue, headache, dizziness and dry mouth) that were reported during the treatment sessions. It also shows the degree of discomfort experienced and the weeks during the treatment period that these side effects occurred. With few exceptions, degree of discomfort was usually rated at 3 or less on a 10-point scale where 0 indicated no discomfort and 10 indicated maximum discomfort. Six participants, including two participants who received sham tDCS, reported increasing tingling and itchiness under the electrode during the initial weeks of the treatment. For one participant (#8), the tingling occurred in more than half of the treatment sessions, reportedly increasing in frequency and magnitude during weeks four, five and six, and extending down her right arm to her fingers; however, overall the degree of discomfort averaged a 3 on the 10-point scale. Fatigue was another side effect that several participants reported, but it is not clear whether these reports were related to the tDCS or other factors. For example, Participant 1 reported excessive tiredness during weeks four to six, which was rated a 2 or 3 on the 10-point scale and coincided with the start of a new blood pressure medication (i.e., Lisinopril) for which excessive tiredness is a noted side effect.

### 3.2. Language Outcome Measures 

Gains in standardized language outcome measures are first reported by group (Table 3) and subsequently for individuals (Table 4). Mean percent gains in oral reading accuracy and rate are reported by group in Table 5. Individual gains in oral reading performance at post-treatment and follow-up are reported in Table 6.

#### 3.2.1. Primary Outcome Measure (WAB-R AQ)

The three groups did not significantly differ in WAB-R AQ scores at baseline, *F*(2,9) = 0.002, *p* = 0.998. Overall WAB-R AQ scores demonstrated significant gains at post-treatment, *F*(1,9) = 13.987, *p* = 0.005, η2 = 0.608; but there was no significant difference in group, *F*(1,9) = 0.002, *p* = 0.998, η2 = 0.000, nor an interaction effect (*p* = 0.985, η2 = 0.003). Significant gains in WAB-R AQ scores were demonstrated for the anodal *t*(3) = 6.107, *p* = 0.009, *d* = 3.06; cathodal groups *t*(3) = 4.416, *p* = 0.022, *d* = 2.21 with large effects sizes, but not for the sham group *t*(3) = 1.255, *p* = 0.298, *d* = 0.63.

Post-hoc power analysis was based on present results, presuming a one-way independent ANOVA given a significance level of *p* = 0.05 and equal treatment allocation, which indicated that 33 participants would be required for a lower powered study (β = 0.2) and 84 participants would be required for a higher (β = 0.5) powered study.

Both the A-tDCS and C-tDCS groups demonstrated a clinically significant improvement (mean gain ≥ 5 points) on the AQ from pre- to post-treatment (5.2 and 5.4, respectively) and from pre-treatment to follow-up (7.4 and 5.7, respectively; Table 3). The mean gains of the S-tDCS group did not meet criteria for clinically significant gains on the AQ for either time interval. At an individual level (Table 4), all four participants receiving A-tDCS, three of four participants receiving C-tDCS, and only one participant receiving S-tDCS stimulation demonstrated clinically significant changes on the AQ from pre-treatment to post-treatment and/or follow-up testing.

#### 3.2.2. Secondary Outcome Measures 

WAB-R Language Quotient. The A-tDCS group demonstrated a clinically significant improvement (mean gain ≥ 5 points) at both time intervals of post-treatment and follow-up; however, the C-tDCS group demonstrated clinically significant changes only at follow-up, not at post-treatment. The S-tDCS group did not meet criteria for clinically significant gains on the LQ for either time interval. At an individual level, all four participants from the A-tDCS group and two of four participants in both the C-tDCS and sham groups made clinically significant changes on the LQ at post-treatment and/or follow-up testing.

CETI. Caregivers of all four participants in the C-tDCS group, two of four participants in the S-tDCS group, and one of three participants in the A-tDCS group reported a clinically significant improvement in communication (mean gain ≥ 10 points) at both post-treatment and follow-up assessments. All other participants in the A-tDCS and S-tDCS groups did not meet criteria for clinically significant gains on the CETI at either time interval. One participant (randomized to A-tDCS) did not have a significant other available to complete the CETI.

Oral Reading Accuracy and Rate. Mean percent gains in oral reading accuracy and rate are reported by group in Table 5. Overall, improvement in oral reading accuracy and rate occurred across all groups. Group means for the percent gain in oral reading accuracy between pre- and post-treatment were greater for the A-tDCS (*M* = 47.3%, SD = 34.7) and C-tDCS (*M* = 47.6%, SD = 37.5) groups than for the S-tDCS group (*M* = 19.9%, SD = 22.8); however, all three groups exhibited a more similar magnitude of gain at follow-up testing. Group means for the percent gain in oral reading rate (wpm) were greater for the A-tDCS and C-tDCS groups than for the S-tDCS group at both post-treatment and follow-up. However, post-hoc non-parametric Kruskal–Wallis tests and Mann–Whitney U tests found no significant group differences on gains in accuracy or rate at post-treatment or follow-up.

Table 6 provides the percent change and effect sizes of NORLA-6 averaged scores for oral reading accuracy and rate for each individual participant. Descriptive analyses of the percent of change on NORLA-6 accuracy and rate scores indicate wide variability across participants. Percent change was typically in the positive direction, except for Participant 12 who received S-tDCS and displayed a negative percent change for accuracy at post-treatment and for rate at both time intervals. Participant 6, who also received S-tDCS, exhibited a negative change for oral reading rate at follow-up testing, while Participant 10, who received C-tDCS, displayed a negative percent change for accuracy at follow-up. Individual effect sizes reaching clinical significance were distributed across all three treatment arms but were more representative of participants with less severe aphasia (AQ > 55) than for those with more severe aphasia (AQ < 55).

### 3.3. fMRI Cortical Activation Patterns

Table 7 provides the neurophysiological data for each participant. With regard to perilesional activation, the three participants (1, 9, and 11; each within a separate treatment arm) with the shortest time post-onset in the sample (range 6.2 to 6.3 months) did not demonstrate increased perilesional activation at post-treatment for any fMRI task. In contrast, the three participants (8, 10, and 12; each within a separate treatment arm) who demonstrated increased perilesional activity across all tasks presented with time post-onset of 7.3, 9.2, and 18.6 months, respectively. The remaining six participants demonstrated increased perilesional activity only on some, but not all tasks ranged from 29.2 to 155.7 months post-onset.

Participants with less severe aphasia tended to exhibit increased activation of the left hemisphere from pre- to post-treatment relative to the right hemisphere, more so than those with more severe aphasia. The oral reading task (Task 2) and the imitation task (Task 3) were more likely to demonstrate increased activation in the left hemisphere from pre- to post-treatment relative to the right hemisphere, than the categorization task (Task 1).

## 4. Discussion

This study recorded stable vitals and no serious adverse events or unintended effects during, or up to six weeks after, an extended application of 1 mA tDCS applied to perilesional cortices for stroke survivors with nonfluent, chronic aphasia. These exploratory results are the first cohort study supporting the safety and tolerability of prolonged tDCS application (i.e., 30 sessions within a six-week interval) in aphasia rehabilitation. The literature has overall reported few adverse events with tDCS delivery, and those that have been reported are relatively mild (e.g., skin irritation, headache, dizziness, and nausea) [10,51,52]. It is notable that the risk of cerebral autoregulation or the reduction in cerebral blood flow following tDCS for patients with cerebrovascular diseases remains unknown [53]; however, recent meta-analyses found no significant difference in the occurrence of adverse events or in dropout rates between active tDCS and control arms in aphasia-focused investigations [10]. Nevertheless, safety and tolerability were important considerations for the present study as the completed protocol was beyond previous maximum durations of tDCS, i.e., 15 sessions within a three-week interval [54] or 14 sessions within an eight-week interval [10,55]. In addition to daily monitoring of vitals and physical side effects within all treatment sessions, we contacted participants weekly during the six-week follow-up interval to ensure that there were no subsequent side effects from the application of 30 doses of tDCS to a specific cortical region. These findings extend our knowledge about the safety limits of tDCS and add to its appeal as an adjuvant to SLT, especially as SLT is typically provided to persons with aphasia over a period of several months rather than isolated two- or three-week intervals.

As the current state of the science lacks consensus on the effectiveness of tDCS to improve communication in persons with aphasia beyond naming, the second aim of this study explored the effects of active tDCS and polarity on language performance and neurophysiology [10]. Specifically, we tested whether A-tDCS or C-tDCS (13 min at 1 mA) delivered concurrently with an evidence-based SLT (90 min daily) for five days a week across six weeks results in greater communication gains and cortical activation than sham stimulation or the opposing polarity. Since participants received SLT within all treatment arms, we expected evident language improvement for all participants, especially in the context of high treatment intensities [9,56]. Beyond the expected gains attributed to behavioral SLT, participants receiving active tDCS (either anodal or cathodal) demonstrated greater gains on standardized language assessments that included all language modalities (auditory comprehension, oral expression, reading comprehension, and writing) than participants receiving sham stimulation. To our knowledge, this may be the first study to report the effects of tDCS on reading comprehension and written expression in aphasia. The same bolstering effect of active tDCS was also seen in caregivers reporting greater, and more frequently clinically significant, gains in daily communication on the CETI for participants receiving active versus sham stimulation. Similarly, participants receiving active tDCS demonstrated nonsignificant but larger gains on the trained oral reading probes than those receiving sham tDCS, although the groups were not matched on baseline performance and individual variability was evident. These results reinforce that tDCS, when paired with SLT, demonstrates potential for amplifying communication gains in persons with aphasia as evidenced across standardized, consumer-reported, and task-specific (i.e., oral reading) outcome measures [10].

The optimal dosage, intensity and frequency of tDCS, and its combination with speech and language therapy is still unclear with great variability across studies that have investigated the adjuvant effects of tDCS on aphasia treatment [10]. With few exceptions, studies have applied between 1 and 2 mA of anodal or cathodal tDCS for 10 to 20 min in 5 to 15 sessions delivered over a period of 1 to 3 weeks [10,13]. In our study we applied 1 mA tDCS for 13 min. As indicated previously, these parameters were selected based on previous studies showing that the effects of 13 min of A-tDCS at 1 mA were maintained for up to 90 min post-stimulation [15]. Nevertheless, a weekly dose of 65 min of tDCS (i.e., 13 min, 5 days/week) is less than the dose provided in many other studies. For example, in the largest double-blinded randomized clinical trial to date that has assessed the efficacy of adjunctive tDCS combined with aphasia therapy, Fridrikkson and colleagues applied 1 mA of anodal tDCS for 20 min over 15 sessions (i.e., 100 min/week for three weeks) [23]. However, when we compare the total dose of tDCS, our study provided 390 min of tDCS over the study period compared to a total of 300 min in the Fridrikkson study.

In addition to the type of behavioral treatment, it is similarly important to consider the dose of the behavioral treatment that is combined with the tDCS. In our study, each session provided 90 min of a combination of ORLA^®^ and AphasiaScripts^®^ for a total of 30 sessions over six weeks compared, for example, to the Fridrikkson study which provided 45 min of a naming treatment for 15 sessions over three weeks [23]. Given the high intensity of the behavioral treatment in our study, and research suggesting that high intensity treatment results in better functional communication outcomes than low intensity treatment [8], it is not possible to dissociate the effects of the tDCS itself from the effects of the behavioral treatment. Indeed, since all groups (anodal tDCS, cathodal tDCS, sham tDCS) received the same intensive behavioral treatment, it is possible that differential effects of the tDCS polarities were masked by the effects of the behavioral treatment, especially on treatment probes that directly measure changes in performance on trained treatment stimuli.

Similar to the current literature, the data from the present study are not sufficient to declare superiority of one polarity versus another in aphasia rehabilitation. Clinically significant gains on the WAB-R AQ and CETI occurred with both polarities at post-treatment and follow-up endpoints; however, clinically significant gains on the CETI were more evident for individuals within the cathodal treatment arm. Clinically significant gains on the WAB-R LQ occurred for both cohorts at follow-up, yet only for the A-tDCS group at post-treatment. Gains in probe measures of oral reading accuracy and rate were noted for both active stimulation groups, with medium effect sizes achieved for some, but not all of the measures. Additionally, fMRI activation patterns demonstrated important polarity effects. C-tDCS to the left, lesioned hemisphere, which was expected to have an inhibitory effect, actually resulted in an increased number of activated voxels within both hemispheres and increased activation in perilesional regions. To date, investigations of cortical stimulation have largely supported a model of interhemispheric inhibition, even though some evidence suggests that unilateral injury can lead to cortical disinhibition, not only in the contralesional homotopic areas connected via the corpus callous, but also in neighboring ipsilesional cortices [57,58,59].

Notably, there was some inconsistency in the direction of change for the number of voxels activated across participants in the present study, with interesting trends of perilesional activation in relation to time post-stroke onset. Thus, supporting that bi-hemispheric or bimodal balance is dynamic even in chronic aphasia (>6 months post-stroke) and is critical for functional recovery in aphasia, stimulation needs to be optimized for the individual patient [18]. Previous research suggests the activation of hemispheric and bihemispheric networks is a dynamic process that changes during the course of stroke recovery and is mediated by several factors including time from aphasia onset and specific task demands [60,61,62]. Typically, in the subacute phase, the right hemisphere exhibits stronger involvement in language functions with a subsequent shift towards increased left hemisphere activation in an apparent attempt to regain dominance within the chronic phase [63,64,65]. We postulate that the relative dominance of the right and left hemisphere roles was still evolving in the participants who were just beyond 6 months post-onset, and thus the window of opportunity to shift activation back to the left hemisphere had not yet opened and precluded the up-regulation of perilesional activation regardless of the treatment provided.

We also suggest that C-tDCS may not always serve an inhibitory role, and in some instances, may be excitatory like A-tDCS. For example, Batsikadze and colleagues compared the effects of 1 mA and 2 mA tDCS on cortical excitability when using 35 cm^2^ electrodes [66]. They found that 1 mA cathodal tDCS decreased corticospinal excitability, whereas 2 mA cathodal tDCS resulted in a significant increase of motor evoked potential amplitudes. While an enhancement of tDCS intensity does not necessarily increase efficacy of stimulation, it may shift the direction of excitability and this should be considered when using different intensities and durations of tDCS [66]. Although we administered 1 mA of C-tDCS in our study, we used a smaller sized electrode (16 cm^2^). Smaller electrodes may produce more focal current density and could lead to more effective and localized neural modulation than larger ones [67,68]. In our study, the smaller electrodes resulted in a current density of 0.063 mA/cm^2^ that more closely resembled, and even exceeded, the current density of a 35 cm^2^ electrode and 2 mA of C-tDCS (i.e., 0.057 mA/cm^2^), thereby possibly shifting the polarity from inhibitory to excitatory.

Long-term potentiation and the maintenance of behavioral treatment gains are critical for aphasia rehabilitation. Across all three standardized measures (WAB-R AQ, WAB-R LQ, and CETI), both conditions receiving active tDCS (anodal and cathodal) continued to exhibit clinically significant gains compared to baseline performance following a six-week hiatus from treatment. Lasting improvements in language may suggest that tDCS in conjunction with brain-derived neurotrophic factor (BDNF) plays a role in enhanced neural plasticity, with more robust long-term learning and efficient reorganization of neural circuits [69,70]. Future research is required to investigate the neurophysiological mechanisms contributing to long-term potentiation during aphasia recovery, and specifically which of those mechanisms are responsive to tDCS, increase synaptic efficacy, and reestablish healthy interhemispheric interactions [69,71,72,73].

We have reported here 12 participants who were randomized to receive anodal, cathodal, or sham stimulation who differed on many variables including lesion size and location. The heterogeneity of lesion and aphasia characteristics are well-known in stroke rehabilitation fields; however, they serve as limitations for study recruitment and generalization of the present findings. To address heterogeneity, a neuronavigation system was used to individualize electrode placement and optimize the effects of the tDCS; however, it resulted in quite disparate electrode placement sites. It is not clear whether or not the fMRI-guided localization of stimulation site served to normalize the effects of the tDCS across individuals. Furthermore, recent computational models have illustrated the complexity of factors contributing to the distribution of current flow through the brain, which may have added further, and at this time indistinguishable, variance into our outcome measures [12,21,22,74,75,76]. Future research may seek to further isolate or account for potentially mediating variables such as time post-aphasia onset by defining more narrow windows of recovery and by more closely matching subjects with regard to lesion location and size.

## 5. Conclusions

The results support that tDCS (delivered to the perilesional cortex at 1 mA for 13 min, five days a week for six weeks) is safe and well tolerated. Further investigation of prolonged, programmatic applications of tDCS combined with behavioral SLT to support aphasia recovery in stroke survivors is warranted based on demonstrated safety, improved language compared to sham stimulation, and increased cortical activation.

## Figures and Tables

**Table 1:** Demographics, clinical characteristics, tDCS allocation, and stimulation site for study participants.

Participant	Age (Years)	Sex	Lesion Size (mL)	TPO (Months)	Educ (Years)	AQ	LQ	CQ	SS	Comp	Rpt	Nmg	Polarity vs. Sham	Stimulation Site
**WAB-R AQ > 55**														
1 JONRA	46.1	M	66.9	6.3	16	70.3	64.0	70.9	12	8.0	6.6	8.6	A-tDCS	MFG
2 SHAER	51.5	M	80.3	29.2	16	61.3	61.9	69.7	10	9.1	4.7	6.9	A-tDCS	FP
3 LEELO	57.1	F	92.8	38.9	17	74.3	74.8	76.2	14	8.8	7.4	7.0	C-tDCS	FP
4 TONMA	57.8	F	94.7	50.9	16	63.9	71.1	73.5	12	9.4	5.7	4.9	C-tDCS	M1
5 PEACA	55.3	F	76.6	155.7	12	70.1	65.1	69.8	12	8.7	7.3	7.1	S-tDCS	MFG
6 PIWTO	61.4	M	155.8	53.3	13	75.3	65.8	70.3	13	8.1	9.5	7.1	S-tDCS	SMG
**WAB-R AQ < 55**														
7 ANDJA	46.1	M	118.6	35.6	12	45.2	46.4	54.2	8	5.5	4.8	4.3	A-tDCS	MFG
8 HOWSH	55.7	F	136.4	9.2	17	47.1	37.4	42.2	9	6.1	5.6	2.9	A-tDCS	MFG
9 KARYA	58.8	M	46.5	6.2	16	38.7	37.6	45.8	9	5.2	4.1	1.1	C-tDCS	IFG
10 TRAWI	64.9	M	83.3	18.6	18	44.0	58.0	61.6	7	8.3	2.8	3.9	C-tDCS	IFG
11 KANJO	71.1	M	24.5	6.2	16	54.3	60.3	65.6	7	7.6	4.8	7.8	S-tDCS	MFG
12 BUTFR	54.7	M	56.1	7.3	18	22.3	30.0	38.4	4	4.8	1.9	0.5	S-tDCS	M1

Note. TPO = Time Post Onset, Educ = Education, WAB-R = Western Aphasia Battery-Revised [25], AQ = Aphasia Quotient, LQ = Language Quotient, CQ = Cortical Quotient, SS = Spontaneous Speech Score (maximum = 20), Comp = Auditory Verbal Comprehension (maximum 10), Rpt = Repetition (maximum = 10), and Nmg = Naming and Word Finding (maximum = 10). A-tDCS = anodal, C-tDCS = cathodal, and S-tDCS = sham. IFG = inferior frontal gyrus, MFG = middle frontal gyrus, FP = frontal pole, M1 = precentral gyrus, and SMG = supramarginal gyrus.

**Table 2:** Reported side effects for each participant.

		Side Effect														
		Tingling/Itching			Fatigue			Headache			Dizzy			Dry Mouth		
ID	Arm *	Number of Sessions ^1^	Avg ^2^	Week ^3^	Number of Sessions	Avg	Week	Number of Sessions	Avg	Week	Number of Sessions	Avg	Week	Number of Sessions	Avg	Week
1	A	6	2.2	1–2	6	2.9	4–6	-	-	-	-	-	-	1	1.0	1
2	A	3	5.3	1	7	3.0	3–5	-	-	-	1	1.0	3	-	-	-
3	C	-	-	-	-	-	-	-	-	-	-	-	-	-	-	-
4	C	-	-	-	-	-	-	-	-	-	-	-	-	-	-	-
5	S	-	-	-	-	-	-	-	-	-	-	-	-	-	-	-
6	S	-	-	-	-	-	-	-	-	-	-	-	-	-	-	-
7	A	-	-	-	10	4.1	2–6	-	-	-	1	2.0	1	-	-	-
8	A	24	3.0	1–6	-	-	-	1	1.5	4	-	-	-	-	-	-
9	C	13	4.6	1–5	5	2.0	1,3,4	1	2.0	3	13	2.0	2–6	-	-	-
10	C	-	-	-	3	1.9	1,2,4	2	1.5	1,4	-	-	-	-	-	-
11	S	6	1.0	1–3	2	6.0	1,6	1	2.0	1	-	-	-	2	1.0	1,3
12	S	14	3.4	1–3	-	-	-	-	-	-	-	-	-	-	-	-

*Arm = Subjects randomized to Anodal (A), Cathodal (C), or Sham (S) tDCS. ^1^ Number of sessions in which side effect was reported out of a total of 30 treatment sessions; ^2^ Average rating on scale of 0–10 with 0 = no discomfort and 10 = maximum discomfort; ^3^ Weeks in which side effect was reported.

**Table 3:** Mean (Std Dev) gains in language performance on the Western Aphasia Battery-Revised (WAB-R) and caregiver-reported communication effectiveness by group at both post-treatment and six-week follow-up.

Group	Number of Participants	AQ Pre-Post	AQ Pre-F	LQ Pre-Post	LQ Pre-F	CETI Pre-Post	CETI Pre-F
A-tDCS	4	5.2 (1.7)	7.4 (2.2)	5.7 (1.8)	6.6 (1.4) ^1^	13.9 (13.3) ^1^	14.5 (17.6) ^1^
C-tDCS	4	5.4 (2.5)	5.7 (5.6)	3.7 (2.6)	6.2 (2.6)	17.2 (2.3)	20.8 (2.1)
S-tDCS	4	4.8 (7.7)	3.1 (8.4)	4.6 (2.2)	3.3 (4.0)	7.2 (6.3)	10.2 (8.0)

^1^ Descriptive statistics were calculated only on three participants Note. Western Aphasia Battery-Revised (WAB-R) [25] AQ = Aphasia Quotient and LQ = Language Quotient. CETI = Communication Effectiveness Index as rated by a caregiver [38]. Pre-Post = gain from pre-treatment to post-treatment, and Pre-F = gain from pre-treatment to follow-up. NA = not available. A-tDCS = anodal, C-tDCS = cathodal, and S-tDCS = sham.

**Table 4:** Individual scores and gains in language performance per the WAB-R (AQ and LQ) and caregiver-reported gains on the CETI.

Participant	Polarity vs. Sham	AQ Pre	AQ Post	AQ F	AQ Pre-Post	AQ Pre-F	LQ Pre	LQ Post	LQ F	LQ Pre-Post	LQ Pre-F	CETI Pre	CETI Post	CETI F	CETI Pre-Post	CETI Pre-F
**WAB-R AQ > 55**																
1	A-tDCS	70.3	76.6	77.1	6.3	6.8	64.0	67.8	69.7	3.8	5.7	47.6	76.9	82.3	29.3	34.7
2	A-tDCS	61.3	68.3	71.7	7.0	10.4	61.9	66.4	70.1	4.5	8.2	33.3	38.9	39.8	5.7	6.6
3	C-tDCS	74.3	77.7	75.5	3.4	1.2	74.8	77.4	79.6	2.6	4.8	45.9	60.2	65.8	14.3	19.9
4	C-tDCS	63.9	67.6	71.5	3.7	7.6	71.1	72.1	74.4	1.0	3.3	54.8	71.3	75.1	16.4	20.3
5	S-tDCS	70.1	72.7	69.5	2.6	-0.6	65.1	67.9	63.2	2.8	-1.9	53.8	54.9	55.9	1.1	2.1
6	S-tDCS	75.3	73.7	71.4	-1.6	-3.9	65.8	69.7	68.1	3.9	2.3	47.6	50.6	54.3	3.0	6.8
**WAB-R AQ < 55**																
7	A-tDCS	45.2	49.1	52.2	3.9	7.0	46.4	53.4	52.2	7.0	5.8	70.1	76.9	72.4	6.9	2.3
8	A-tDCS	47.1	50.7	52.4	3.6	5.3	37.4	44.8	NA	7.4	NA	NA	NA	NA	NA	NA
9	C-tDCS	38.7	44.5	39.8	5.8	1.1	37.6	44.4	46.6	6.8	9.0	70.4	88.8	94.3	18.3	23.9
10	C-tDCS	44.0	52.7	56.8	8.7	12.8	58.0	62.4	65.8	4.4	7.8	44.2	63.8	63.4	19.6	19.2
11	S-tDCS	54.3	70.3	69.6	16	15.3	60.3	68.1	66.8	7.8	6.5	24.7	39.1	45.7	14.4	21.0
12	S-tDCS	22.3	24.6	24.4	2.3	1.9	30.0	33.9	36.3	3.9	6.3	38.2	48.7	48.4	10.5	10.2

Note. Western Aphasia Battery-Revised (WAB) [25] AQ = Aphasia Quotient and LQ = Language Quotient. CETI = Communication Effectiveness Index as rated by a caregiver [38] Pre = Pre-treatment. Score, Post = post-treatment score, F = follow-up score, Pre-Post = gain from pre-treatment to post-treatment, and Pre-F = gain from pre-treatment to follow-up. NA = not available. A-tDCS = anodal, C-tDCS = cathodal, and S-tDCS = sham.

**Table 5:** Mean percent gains (Std Dev) in oral reading accuracy and rate by group at both post-treatment and six-week follow-up testing.

Group	Number of Participants	Oral Reading Accuracy Gain (SD) at Post- Treatment	Oral Reading Accuracy Gain (SD) at Follow-Up Testing	Oral Reading Rate Gain (SD) at Post-Treatment	Oral Reading Rate Gain (SD) at Follow-Up Testing
A-tDCS	4	47.3 (34.7)	25.7 (0.6) ^1^	63.2 (62.8)	120.7 (74.3) ^1^
C-tDCS	4	47.6 (37.5)	30.2 (47.3)	43.7 (53.4)	140.4 (96.3)
S-tDCS	4	19.9 (22.8)	32.5 (12.7)	21.4 (55.2)	15.8 (41.6)

^1^ Calculated only on two participants. Participant 8 refused to complete the probes; Participant 2 completed the probes, but they could not be scored because of failed recording equipment. Note: Percent gain calculated as the mean of three post-treatment probes or the mean of two 6-week follow-up probes minus the mean of three pre-treatment probes and subsequently divided by the mean of the three baseline probes. A-tDCS = anodal, C-tDCS = cathodal, and S-tDCS = sham.

**Table 6:** Individual scores and percent gain (effect size) in oral reading accuracy and rate (wpm) per the Naming and Oral Reading for Language in Aphasia 6-point scale (NORLA-6).

Participant	Polarity vs. Sham	Oral Reading Accuracy Gain (ES) at Post-Treatment	Oral Reading Accuracy Gain (ES) at Follow-Up Testing	Oral Reading Rate Gain (ES) at Post-Treatment	Oral Reading Rate Gain (ES) at Follow-Up Testing
**WAB-R AQ > 55**					
1	A-tDCS	36.2 (5.2)	26.0 (3.8)	58.0 (4.5)	28.2 (2.2)
2	A-tDCS	91.5 (8.1)	NA	47.7 (9.5)	NA
3	C-tDCS	17.2 (2.3)	20.1 (2.7)	15.8 (2.2)	134.7 (18.9)
4	C-tDCS	89.5 (4.8)	92.0 (5.0)	13.7 (0.7)	110.3 (5.0)
5	S-tDCS	40.0 (6.0)	27.8 (4.2)	67.7 (11.7)	50.5 (8.8)
6	S-tDCS	26.6 (4.2)	21.2 (3.4)	55.1 (11.2)	−27.3 (−5.5)
**WAB-R AQ < 55**					
7	A-tDCS	8.7 (0.4)	25.2 (1.0)	−5.0 (0.0)	455.0 (14.2)
8	A-tDCS	52.5 (5.8)	NA	146.7 (6.0)	NA
9	C-tDCS	14.5 (0.7)	30.8 (1.6)	124.0 (6.7)	353.6 (19.0)
10	C-tDCS	69.3 (2.4)	−22.5 (−0.8)	20.8 (0.4)	31.3 (0.6)
11	S-tDCS	25.7 (2.4)	30.1 (2.8)	16.5 (2.2)	1.2 (0.2)
12	S-tDCS	−13.0 (−5.4)	50.4 (21.2)	−54.0 (−1.6)	−54.0 (−1.6)

Note. Participant 8 refused to complete the follow-up probes; Participant 2 completed the follow-up probes but they could not be scored because of failed recording equipment. Percent gain calculated as the mean of three post-treatment probes or the mean of two 6-week follow-up probes minus the mean of three pre-treatment probes and subsequently divided by the mean of the three baseline probes. ES = effect size, calculated as the mean of three post-treatment probes or the mean of two 6-week follow-up probes minus the mean of three pre-treatment probes and subsequently divided by the standard deviation across the three baseline probes [42]. A clinically significant effect size was determined apriori to be greater than 3.9 (medium effect) [44,45]. NA = not available. A-tDCS = anodal, C-tDCS = cathodal, and S-tDCS = sham.

**Table 7:** Number of activated voxels within a 5 mm area around the lesion (perilesional) and the ratio of activated voxels between the left vs. right hemisphere (left/right ratio) at pre- and post-treatment.

Participant	Polarity vs. Sham	Lesion Size (mL)	Perilesional Activation						Left/Right Ratio					
			Task 1		Task 2		Task 3		Task 1		Task 2		Task 3	
			Pre	Post	Pre	Post	Pre	Post	Pre	Post	Pre	Post	Pre	Post
**WAB-R AQ > 55**														
1	A-tDCS	66.9	57	0	0	0	135	122	0.74	0.56	0.91	0.76	0.56	0.86
2	A-tDCS	80.3	339	92	86	6	142	284	0.75	0.71	0.68	4.31	0.47	0.70
3	C-tDCS	92.8	816	375	45	2151	103	2244	0.68	0.75	0.51	1.53	0.81	1.60
4	C-tDCS	94.7	34	81	317	114	357	434	0.76	0.86	0.35	0.66	0.58	0.65
5	S-tDCS	76.6	768	641	181	117	216	600	0.45	0.69	0.35	0.44	0.53	0.71
6	S-tDCS	155.8	238	238	24	0	154	412	0.67	0.43	0.12	0.17	0.39	0.81
**WAB-R AQ < 55**														
7	A-tDCS	118.6	227	467	NA	NA	NA	NA	0.89	0.94	NA	NA	NA	NA
8	A-tDCS	136.4	234	494	62	670	48	539	0.97	0.67	0.84	1.67	0.58	0.80
9	C-tDCS	46.5	121	28	0	36	255	43	0.60	0.78	0.73	0.28	0.64	0.32
10	C-tDCS	83.3	98	139	19	71	96	147	0.91	0.85	1.02	0.74	1.07	0.93
11	S-tDCS	24.5	57	0	0	0	135	122	1.27	1.00	0.78	1.08	1.12	0.80
12	S-tDCS	56.1	0	88	205	380	232	292	0.12	1.76	0.25	2.56	0.83	5.09

Note. For Participant 7, tasks 2 and 3 resulted in a large number of movement artefacts at both pre- and post-treatment so these tasks were subsequently excluded from analysis. WAB-R AQ = Western Aphasia Battery-Revised Aphasia Quotient [25]. Pre = pre-treatment score, Post = post-treatment score. A-tDCS = anodal, C-tDCS = cathodal, and S-tDCS = sham. For Left/Right Ratio: a value of 1 indicates equal activation in left vs. right hemisphere, a value > 1 indicates more activity in the left hemisphere, and a value < 1 indicates more activity in the right hemisphere. For perilesional activation, highlighted cells indicate increased perilesional activation at post-treatment. For left/right ratio, highlighted cells indicate increased left hemisphere activity from pre- to post-treatment.
